# Pediatric advance care planning: a mixed-methods evaluation of documentation and sharing in current practice

**DOI:** 10.1186/s12904-026-01992-7

**Published:** 2026-01-29

**Authors:** Sophie Tooten, Rosella P.M.G. Hermens, Manel Verhoeven, Ellen M. Vierhoven, Fatima Boulakhrif, Jana M. Reintjes, Michel A.A.P. Willemsen, Judith L. Aris-Meijer, Jurrianne C. Fahner, Marijanne Engel, Marijke C. Kars, Inge M.L. Ahout, Esther Deuning-Smit

**Affiliations:** 1 Department of Pediatrics, Radboud University Medical Center, Amalia Children’s Hospital, Nijmegen, Netherlands; 2 Science Department IQ Health, Radboud University Medical Center, Nijmegen, Netherlands; 3 University Medical Center Groningen, Beatrix Children’s Hospital, Groningen, Netherlands; 4 University Medical Center Utrecht, Wilhelmina Children’s Hospital, Utrecht, Netherlands; 5 Julius Center for Health Sciences and Primary Care, Center of Expertise in Palliative Care Utrecht, University Medical Center Utrecht, Utrecht, Netherlands

**Keywords:** Pediatrics, Palliative care, Advance care planning, Information dissemination, Interprofessional collaboration, Documentation, Goals of care, Electronic health record documentation

## Abstract

**Background:**

Pediatric advance care planning (pACP) aims to align future care and treatment of children with life-limiting conditions (LLCs) with children’s and their families’ values, goals and preferences. Documenting and sharing pACP elements with healthcare professionals (HCPs) is essential for goal-concordant care. This study evaluates how pACP elements are documented in electronic health records of children with LLCs and shared with other HCPs.

**Methods:**

A mixed-method study was conducted at a Dutch academic children’s hospital. A retrospective medical chart review examined electronic health records of all children with LLCs who died in 2023, assessing patient characteristics, disease and care characteristics, and pACP elements dating back to the first hospital visit. Structured interviews with primary physicians explored their perspectives on the occurrence, documentation and sharing of pACP in these patients.

**Results:**

Eleven thousand, three hundred and thirty notes were screened for 28 patients, written by different HCP types. pACP elements were identified in 127 notes (1%) from 16 patients (57%). Documentation occurred most often during a phase of health deterioration (70%). The most frequently documented pACP element was ‘goals of future care and treatment’ (78%), first recorded a median of 2 years after diagnosis, while ‘preferred location of death’ was least documented (38%). Of the four palliative care domains (physical, psychological, social and existential), the physical domain was most commonly addressed (48%). Individual patient timelines showed three groups: patients without pACP documentation, those with pACP documentation at the end of life, and those with pACP documentation throughout treatment duration. Inter-organizational sharing of pACP elements was rarely documented (26%). Primary physicians (*N* = 7) reported discussing and sharing pACP elements more often than documenting them. Reasons for under-documentation included avoidance of repetition, unclear responsibility, perceived irrelevance for others, and difficulty capturing complex conversations.

**Conclusion:**

Implementation of pACP in pediatric palliative care could be improved. Documentation does not fully reflect actual care, as physicians reported sharing more pACP information with other HCPs both intra-and inter-organizationally than reflected in patient records. Several barriers to documenting and sharing were mentioned, and future studies should focus on developing tools and guidelines to support HCPs in improving documentation and sharing of pACP processes and outcomes.

**Supplementary Information:**

The online version contains supplementary material available at 10.1186/s12904-026-01992-7.

## Introduction

Children diagnosed with life-limiting conditions (LLCs), together with their families, often face significant uncertainty about the future [1–3]. For children, concerns about how the illness will affect meaningful activities and their participation in everyday life often outweighs concerns about the illness itself [4]. While hope for a favorable outcome remains, there is also shared awareness of the importance of preparing for unfavorable scenarios such as disease progression, decrease in quality of life, or even a child’s death [1, 5–8].

Pediatric advance care planning (pACP) supports families as they navigate this uncertainty by identifying values, goals and preferences for future care and treatment with their healthcare professional (HCP) and, when necessary, translating these into concrete actions and agreements [6, 9, 10]. Early initiation of pACP is recommended for HCPs working in pediatric palliative care [11–13], with ongoing refinement as an iterative process tailored to the course of the child’s disease and coping mechanisms of parents and children. pACP is a holistic approach in which the four domains of palliative care (physical, psychological, social, and existential) form the foundation for exploring goals and preferences for future care and treatment. The recent international pediatric palliative care standard [13] lists additional topics for pACP discussions including provision of information on diagnosis and prognosis, emergency planning for life-sustaining treatment and other acute scenarios, and preferences for the place of end-of-life care and death [13].

Aligning actual care to goals and preferences clarified in pACP conversations is challenging, due to the involvement of multiple HCPs working across different organizations and who are involved in different points in the child’s illness trajectory. A significant challenge is to ensure that all involved HCPs are informed about family’s values, goals and preferences, which can be facilitated by either HCPs or parents. For HCPs, documenting pACP conversations in the patient’s electronic health record is a first step to sharing this information with the care network. It should be noted that when the responsibility for information sharing is placed on the parents this may result in a significant burden [13].

While national and international guidelines and standards emphasize the importance of documenting and sharing pACP elements with other involved HCPs [12–14], the recommendations are not uniform. The revised Dutch guideline for pediatric palliative care (2022) [14] includes a comprehensive chapter on pACP, accompanied by a concise section on ‘documentation and sharing’. It emphasizes the documentation of the content of pACP discussions within patients’ electronic health records and Individual Care Plans (a comprehensive document capturing preferences, desires and agreements regarding current and future care of the child) [15, 16], followed by sharing specific treatment agreements with all HCPs involved. In contrast, the recently published international ‘Global Overview – Pediatric Palliative Care Standards’ (GO-PPaCS) [13] is more general, advising to document concise recommendations for emergency settings and end-of-life care in an ACP document, without specifying the content of these recommendations. The guideline from the National Institute for Health and Care Excellence (NICE) is the most comprehensive [12], recommending the development of an advance care plan for each patient, and providing an outline of what should be included in this plan (e.g. child’s ambitions and wishes, a summary of the condition and agreed treatment plans).

The variation among these guidelines raises the question of how pACP elements obtained from conversations with the child or family are currently documented and shared with other HCPs. To the best of our knowledge, no previous study has evaluated this. Therefore, this study aims to evaluate the extent to which documentation and sharing of pACP elements with other HCPs is implemented in current practice for children with LLCs.

## Methods

### Study design

**Fig. 1:**
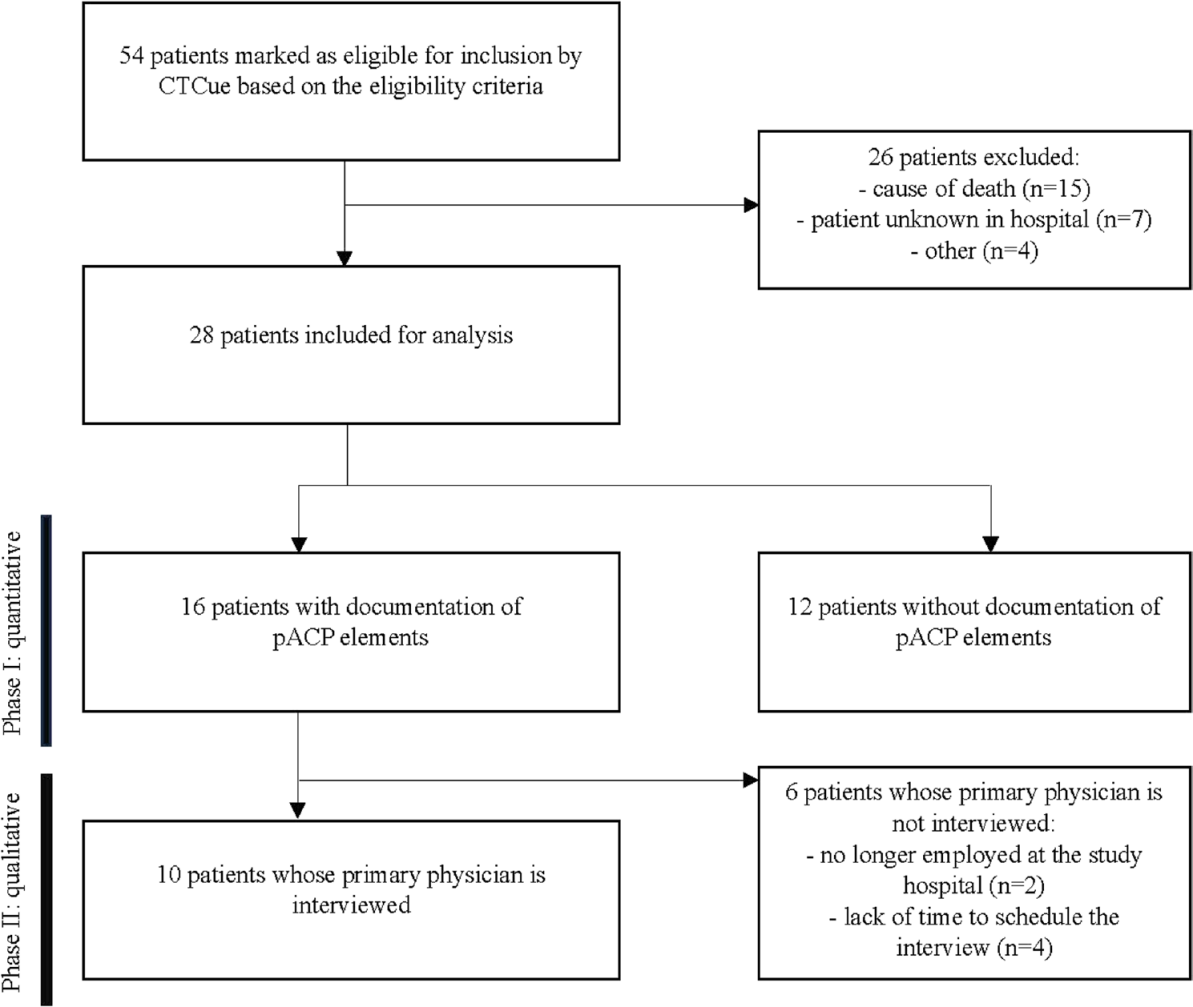
Study flow diagram

We conducted a mixed-method study using the two-phase Explanatory Sequential Design, with a quantitative component followed by a qualitative component [17]. Quantitative data collection comprised a retrospective medical chart review, conducted at a Dutch academic children’s hospital, hereafter referred to as the ‘study hospital’ in this article. In order to gain a deeper understanding of these quantitative results, structured interviews with the primary physicians were conducted. Figure 1 shows an overview of the study design. Institutional review board approval was requested and waived under protocol number 2023–16,933. The requirement of informed consent was waived due to the retrospective nature of the study.

### Setting

In 2023, approximately 1,000 children (aged 0–20 years) died in the Netherlands [18]. Of these, around 500 deaths were attributed to perinatal causes. Among the remaining 500 deaths, 150 were due to external causes, such as traffic accidents, and 70 were attributed to cancer, with all cancer cases being treated at a specialized pediatric oncology center in the Netherlands.

The study hospital is one of seven academic children’s hospitals in the Netherlands and covers the south-eastern part of the country, treating around 26,000 unique patients annually. It provides a wide range of tertiary pediatric services, including pediatric and neonatal intensive care. All Dutch academic children’s hospitals have a pediatric palliative care team, providing expertise, case managers and support in pACP during hospital admissions, outpatient clinic visits, and also at home.

There has been significant progress in the implementation of pACP within pediatric care in the Netherlands. The focus is shifting from end-of-life care to a broader, earlier integration of pACP that fosters better communication and alignment of care between healthcare providers and families throughout the disease trajectory [19]. In the Netherlands, multiple electronic health record systems are used across different healthcare organization, but there is a notable lack of integration between these systems. Developments aimed at marking and centralizing pACP discussions within the electronic health record system are still in their early stages and have yet to be widely implemented.

### Quantitative component: retrospective medical chart review

#### Study participants

We retrospectively identified eligible patients using text-mining software (CTcue B.V., Amsterdam, the Netherlands). This software program is linked to fields in the hospital’s electronic health records in order to identify eligible patients [20] based on structured key eligibility criteria and free-text medical notes. Patients were eligible if < 18 years old, deceased in 2023, and had a hospital admission or outpatient visit at the study hospital in their medical history. Patients were excluded if they died due to perinatal complications, sudden infant death syndrome, or trauma including abuse.

#### Data collection

Data were extracted from the electronic health records in accordance with a predefined protocol (Supplementary material 1), created in collaboration with pediatricians and senior researchers. The protocol was subjected to a process of testing and adapting, using three electronic health records of patients who died in 2022. The content of the protocol was divided into the following categories: patient characteristics, disease and care characteristics, and pACP elements. One researcher (FB, JR or MV) extracted the patient, disease and care characteristics individually. Patient characteristics included age at diagnosis, age at death and biological sex. Disease and care characteristics included diagnosis, category of LLC, hospital visits, treatment duration (time between first and last contact), involvement of the pediatric palliative care team and location of death. LLCs were categorized as: (I) life-threatening conditions for which curative treatment may be feasible but can fail; (II) conditions where premature death is inevitable; (III) progressive conditions without curative treatment options; and (IV) irreversible but non-progressive conditions causing severe disability and susceptibility to health complications [21]. To define the extent to which death could be anticipated in the disease course, two physicians from the research team (ST and IA) assessed whether the cause of death was in line with the underlying LLC.

To determine the presence of pACP elements, all clinical notes, incoming and outgoing medical correspondence in the patients’ electronic health records, from the date of the first hospital inpatient, outpatient, or emergency room contact, were independently manually reviewed by at least two of the four researchers (ST, FB, JR, MV). Notes and correspondence from physicians, nurses, nurse practitioners, child life specialists, social workers, chaplains, paramedics and psychologists were reviewed. Predefined discussion topics from the international GO-PPaCS standard [13] were used to specify the following pACP elements: provision of information on diagnosis and prognosis, goals of future care and treatments, emergency planning to provide medical orders for life-sustaining treatment, preferred place to receive end-of-life care, and preferred place of the child’s death. ‘Provision of information on diagnosis and prognosis’ was excluded as a standalone pACP element, as it solely involves information delivery from an HCP to the patient and/or their family without discussing values, goals and preferences. However, if this element was discussed in combination with any of the other pACP elements, it was included in the data extraction. The pACP element ‘goals of future care and treatments’ was scored as present if values, goals and/or preferences related to any of the palliative care domains (physical, psychological, social or existential) were documented. The comprehensiveness of documentation of this pACP element was then evaluated, scoring from zero (absent) to three (comprehensive, including specific discussion details). The pACP element ‘emergency planning to provide medical orders for life-sustaining treatment’ was scored as present if possible future scenarios or specific treatment limitations were documented such as agreements on intensive care admission or do-not-resuscitate orders. The pACP elements ‘preferred place to receive end-of-life care’ and ‘preferred place of the child’s death’ were scored as present if any wishes were documented regarding these topics. When a pACP element was identified in a note in the electronic health record, several other characteristics of this note were extracted including date, author of the note, phase of disease (stable, deterioration, terminal) and location of the pACP discussion. The medical correspondence was reviewed to determine if and with whom the documented pACP elements were shared. Documentation of alternative sharing methods, such as telephone or multidisciplinary team meetings, was also assessed.

After screening the electronic health records, the extracted data were reviewed and discussed to reach consensus by two researchers. If consensus could not be reached, a third independent researcher was consulted to reach a final decision. To differentiate notes with pACP elements from those addressing routine daily care for that moment in time, only notes demonstrating a future-oriented perspective were included, since pACP aims to explore patients’ values, goals and preferences concerning future care and treatment rather than ongoing daily care. Data was extracted into CastorEDC, an electronic data capture system used for secure collection and management of research data [22].

#### Data analysis

Descriptive statistics were utilized to represent the patient characteristics (e.g., age at diagnosis and death, sex) and the care processes (e.g., diagnosis, cause of death, hospital visits), as well as the pACP elements. Categorical variables were expressed as counts and percentages, continuous variables as means with standard deviations, and with a skewed distribution as medians with interquartile ranges (IQR).

An individual patient timeline for each patient of their pACP trajectory, from first hospital contact till moment of death, was created in order to provide a visual representation of their course of pACP and facilitate pattern recognition. Subsequently, the research team (ST, IA, ED) reviewed these timelines together in order to identify recurring patterns or similarities between the cases.

### Qualitative component: structured interviews

The qualitative methodology was reported following the COREQ checklist for qualitative research (Supplementary material 2) [23].

#### Study participants

For patients with documented pACP elements, the primary physician was identified through the electronic health record and this was either the regular physician at the outpatient clinic, the primary physician during the last prolonged hospital admission, or explicitly noted as primary physician in the health record. Unclear cases were discussed with a pediatrician working at the study hospital. Identified primary physicians currently employed at the study hospital were invited by e-mail to participate in a structured in-person interview. Prior to the start of the interview, the participants provided written informed consent and completed a questionnaire collecting demographic characteristics.

#### Data collection

The interview guide (Supplementary material 3) was based upon the medical chart review protocol, with the objective of adding context and depth to the findings derived from the medical chart review. The findings of the medical chart review, were presented to the interviewed primary physicians in the form of an individual patient timeline that visualized the pACP trajectory over time, including all notes containing a pACP element. The primary physician was asked by closed-ended questions (e.g., “Looking back on the advance care planning process for this patient and his/her family, did you discuss their goals and preferences for future care and treatment?”) whether they found this timeline recognizable and how they remembered applying pACP within this patient trajectory. If any discrepancies were reported between the HCP’s memory and data derived from the electronic health records, open-ended questions (e.g., “Why is there a difference?”) were posed to explore the underlying cause of this discrepancy. All interviews were conducted in-hospital by the same researcher (EV), who was unfamiliar with the primary physicians, and were audio-recorded using Microsoft Teams. Duration of the interviews varied between 30 and 60 min.

#### Data analysis

From the audio recording, the responses to closed-ended questions were entered and stored into CastorEDC and analyzed using descriptive statistics. The responses to open-ended questions were transcribed verbatim, pseudonymized, stored and coded. Two researchers (EV, medical student and ST, physician and PhD candidate) coded the responses. Both researchers received training in qualitative methods and were closely supervised throughout the process by a more experienced researcher (ED, psychologist and postdoctoral researcher). Responses to each open-ended question were coded separately, following an inductive approach as no pre-defined theory or framework was used. The first three interviews were coded independently by two researchers (EV, ST) and discrepancies in coding were discussed until consensus was reached. For the subsequent interviews, one researcher (EV) reviewed the coding for consistency, and when consensus could not be reached, the second researcher (ST) was consulted. A third researcher (IA or ED) was available if consensus could not be reached, though this was not required. After coding, the researchers further clustered the codes into main themes for each question separately (Supplementary material 4). These themes were discussed amongst the research team members to ensure coherence and alignment with the data. To enhance the trustworthiness of the identified themes, the findings were presented at a research meeting with pediatricians in the study hospital, providing an opportunity for feedback.

## Results

We evaluated to what extent pACP elements are documented and shared among HCPs in current practice. CTcue identified 54 eligible patients based on the eligibility criteria. After screening of their electronic health records by one researcher (ST), a total of 28 patients were included for the medical chart review. Among these, 16 (57%) had documented elements of pACP, with a median of five notes (IQR 2–10) containing at least one pACP element. The primary physicians of these 16 patients were invited to participate in the structured interviews. A total of seven primary physicans participated in the interviews, covering ten patients, as some patients shared the same primary physician (Fig. 1).

### Quantitative component: retrospective medical chart review

#### Patient, disease and care characteristics

Table 1 provides an overview of the patient, disease and care characteristics for patients with and without documented pACP elements. Following a detailed discussion of the individual patient timelines within the research team, three distinct patient groups could be identified: (1) Patients without documented pACP elements (*n* = 12); (2) patients with documented pACP elements predominantly at the end of life (*n* = 7); and (3) patients with documented pACP elements throughout the entire treatment duration in the study hospital (*n* = 9). Patients with documentation of pACP elements throughout the entire treatment duration tended to be younger at the time of diagnosis. In patients without documented pACP elements, the cause of death was less often in line with their LLC (e.g., acute sepsis in a patient with metabolic disease), and they visited the study hospital less often throughout their treatment, particularly in the final year of life. The time between their last hospital contact and death was longer, and the pediatric palliative care team was involved less frequently. When involved, there was a longer period of time between last contact and death.

**Table 1:** Patient, disease and care characteristics

Variable		pACP Group			Total
		No pACP	pACP at the end of life	pACP throughout entire treatment duration	
		*n* = 12	*n* = 7	*n* = 9	*N* = 28
Age at diagnosis, *years*,* median (IQR)*		4 (1–8)	3 (1–7)	1 (0–1)	1 (0–6)
Age at death, *years*,* median (IQR)*		11 (7–14)	11 (4–14)	4 (2–9)	10 (3–14)
Female sex, *n (%)*		8 (66)	3 (43)	5 (56)	16 (57)
Diagnosis					
Neurologic, *n (%)*		3 (25)	4 (57)	5 (56)	12 (43)
Congenital or genetic, *n (%)*		1 (8)	2 (29)	1 (11)	4 (14)
Cardiovascular, *n (%)*		3 (25)	0	1 (11)	4 (14)
Metabolic, *n (%)*		3 (25)	1 (14)	0	4 (14)
Neonatal, *n (%)*		0	0	1 (11)	1 (4)
Renal, *n (%)*		0	0	1 (11)	1 (4)
Malignancy, *n (%)*		1 (8)	0	0	1 (4)
Pulmonary, *n (%)*		1 (8)	0	0	1 (4)
Category of life-limiting condition					
I: Life-threatening conditions for which curative treatment may be feasible but can fail, *n (%)*		3 (21)	0	3 (33)	6 (21)
II: Conditions where premature death is inevitable, *n (%)*		0	0	0	0
III: Progressive conditions without curative treatment options, *n (%)*		4 (33)	2 (29)	2 (22)	8 (29)
IV: Irreversible but non-progressive condition causing severe disability, leading to susceptibility to health, *n (%)*		5 (36)	5 (71)	4 (44)	14 (50)
Cause of death in line with underlying LLC					
Yes, *n (%)*		6 (50)	6 (86)	8 (89)	20 (71)
No, *n (%)*		2 (17)	1 (14)	1 (11)	4 (14)
Cause undocumented, *n (%)*		4 (33)	0	0	4 (14)
Location of death					
Other hospital, *n (%)*		3 (25)	1 (14)	4 (44)	8 (29)
Ward study hospital, *n (%)*		2 (17)	3 (43)	1 (11)	6 (21)
PICU study hospital, *n (%)*		0	3 (43)	1 (11)	4 (14)
Home, *n (%)*		2 (17)	0	2 (22)	4 (14)
Emergency room study hospital, *n (%)*		1 (8)	0	0	1 (4)
Unknown, *n (%)*		4 (33)	0	1 (11)	5 (18)
Treatment duration study hospital					
Time between first and last contact, *years*,* median (IQR)*		5 (2–6)	7 (3–11)	1 (1–4)	4 (1–8)
Time between last contact and death, *days*,* median (IQR)*		185 (14–338)	0 (0)	1 (0–9)	1 (0–54)
Hospital visits study hospital					
Entire treatment duration, *number*, *median (IQR)*		7 (3–13)	29 (23–35)	5 (2–20)	10 (2–25)
Last year of life, *number*,* median (IQR)*		1 (1–2)	8 (6–12)	3 (1–5)	3 (1–5)
Primary physician employed in study hospital, *n (%)*		8 (67)	7 (100)	7 (78)	22 (79)
Pediatric palliative care team					
Involved, *n (%)*		3 (25)	5 (71)	6 (67)	14 (50)
Time between diagnosis and first contact with pediatric palliative care team, *years*,* median (IQR)*		5 (3–6)	7 (4–9)	2 (1–3)	4 (2–7)
Time between last contact with pediatric palliative care team and death, *days*,* median (IQR)*		907 (609–1101)	0 (0–2)	1 (1–4)	2 (0–17)

#### pACP characteristics

Across all electronic health records, a total of 11,330 notes were reviewed of which 127 (1%) contained at least one for pACP element. The characteristics of these notes are shown in Table 2. All patients with documented pACP elements (100%) had at least one note mentioning ‘goals of future care and treatment’, while ‘preferred location for end-of-life care’ and ‘preferred location of death’ were less frequently documented (*n* = 7 patients, 44% and *n* = 6 patients, 38%, respectively). The four domains of palliative care were found dispersed throughout the notes containing pACP elements, with the physical domain being more prevalent (*n* = 61 notes, 48%) than the other domains. In less than half of the patients (*n* = 6 patients, 38%), all palliative care domains were identified at least once. In all four palliative care domains, pACP elements were most often documented with moderate comprehensiveness, meaning that the content was described at a basic level, but lacked detailed information or specific examples. One-third of patients (*n* = 5 patients, 31%) had at least one note with pACP elements in each phase of the disease. The majority of pACP notes (*n* = 89 notes, 70%) were found at a time when the patient was in a phase of deterioration. The timing of initial pACP documentation after diagnosis varied widely, with ‘provision of information on diagnosis and prognosis’ and ‘goals of future care and treatment’ often being documented much earlier (median 1 (IQR 0–4) and 2 (IQR 0–6) years after diagnosis, respectively) than ‘preferred location of death’ (median 5 years after diagnosis (IQR 1-8). The last note containing pACP elements frequently occurred just a few days before death. Notes were found to be documented during admissions to the pediatric ward (*n* = 45 notes, 35%), pediatric intensive care unit (PICU) (*n* = 32 notes, 25%), or during outpatient visits (*n* = 30 notes, 24%). pACP elements were also identified in notes from consultations that occurred at home (*n* = 1 note, 1%), via telephone (*n* = 8 notes, 6%), or via video consultations (*n* = 5 notes, 4%). HCPs discussing pACP elements were predominantly physicians (*n* = 96 notes, 76%), followed by members of the pediatric palliative care team (*n* = 11 notes, 9%) and social workers (*n* = 9 notes, 7%).

**Table 2:** pACP characteristics

	Notes with pACP elements* (*n* = 127)	Patients with pACP** elements (*n* = 16)
pACP elements		
Goals of future care and treatment, *n (%)*	99 (78)	16 (100)
Emergency planning, *n (%)*	44 (35)	12 (75)
Provision of information on diagnosis and prognosis, n (%)	22 (17)	11 (68)
Preferred location of end-of-life care, *n (%)*	11 (9)	7 (44)
Preferred location of death, *n (%)*	12 (9)	6 (38)
Domains of palliative care and comprehensiveness of documentation		
Physical total, *n (%)*	61 (48)	14 (88)
- Minimal, *n (%)*	3 (2)	3 (19)
- Moderate, *n (%)*	40 (41)	12 (75)
- Comprehensive, *n (%)*	18 (14)	9 (56)
Psychological total, *n (%)*	31 (24)	12 (75)
- Minimal, *n (%)*	3 (2)	2 (13)
- Moderate, *n (%)*	23 (18)	10 (63)
- Comprehensive, *n (%)*	5 (4)	4 (25)
Social total, *n (%)*	30 (23)	12 (75)
- Minimal, *n (%)*	1 (1)	1 (6)
- Moderate, *n (%)*	24 (19)	10 (63)
- Comprehensive, *n (%)*	5 (4)	5 (31)
Existential total, *n (%)*	34 (27)	10 (63)
- Minimal, *n (%)*	1 (1)	1 (6)
- Moderate, *n (%)*	27 (21)	10 (63)
- Comprehensive, *n (%)*	6 (5)	2 (13)
All domains, *n (%)*	1 (1)	6 (38)
Phase of illness		
Stable, *n (%)*	15 (12)	5 (31)
Deterioration, *n (%)*	89 (70)	14 (88)
Terminal, *n (%)*	23 (18)	12 (75)
All phases, *n (%)*	N/A	5 (31)
Time between diagnosis and first ACP element		*Median time between diagnosis and* *first* *note*
Provision of information on diagnosis and prognosis, *years*,* median (IQR)*		1 (0–4)
Goals of future care and treatment, *years*,* median (IQR)*		2 (0–6)
Emergency planning, *years*,* median (IQR)*		3 (2–6)
Location of end-of-life care, *years*,* median (IQR)*		4 (1–6)
Location of death, *years*,* median (IQR)*		5 (1–8)
Time between last ACP element and death		*Median time between* *last* *note and death*
Provision of information on diagnosis and prognosis, *days*,* median (IQR)*		71 (13–205)
Goals of future care and treatment, *days*,* median (IQR)*		6 (1–10)
Emergency planning, *days*,* median (IQR)*		18 (4–48)
Location of end-of-life care, *days*,* median (IQR)*		7 (5–48)
Location of death, *days*,* median (IQR)*		6 (4–44)
Location of conversation		
Pediatric ward, *n (%)*	45 (35)	9 (56)
PICU, *n (%)*	32 (25)	12 (75)
Outpatient clinic, *n (%)*	30 (24)	9 (56)
Emergency room, *n (%)*	3 (2)	3 (19)
Phone call, *n (%)*	8 (6)	6 (38)
Home, *n (%)*	1 (1)	1 (6)
Video, *n (%)*	5 (4)	4 (25)
Unknown, *n (%)*	3 (2)	3 (19)
HCP discussing pACP elements		
Physician, *n (%)*	96 (76)	16 (100)
Pediatric palliative care team member, *n (%)*	11 (9)	7 (44)
Social worker, *n (%)*	9 (7)	2 (13)
Nurse, *n (%)*	4 (3)	5 (31)
Childe life specialist, *n (%)*	4 (3)	2 (13)
Nurse practitioner, *n (%)*	2 (2)	2 (13)
Psychologist, *n (%)*	1 (1)	1 (6)
Sharing of pACP elements		
Medical letter, *n (%)*	28 (22)	12 (75)
Phone call, *n (%)*	5 (4)	5 (31)
Other, *n (%)*	0	0
Not shared, *n (%)*	96 (76)	2 (13)
Comprehensiveness in letter compared to note		
Less, *n (%)*	14 (50)	8 (50)
Comparable, *n (%)*	9 (32)	5 (31)
More, *n (%)*	5 (17)	4 (25)
Recipients of medical letter		
*Intra-organizational*		
Medical specialist, *n (%)*	12 (43)	4 (25)
Pediatric palliative care team member, *n (%)*	0	0
Social worker, *n (%)*	0	0
Child life specialist, *n (%)*	0	0
Paramedics, *n (%)*	0	0
*Inter-organizational*		
General practitioner, *n (%)*	25 (89)	12 (75)
Regional pediatrician, *n (%)*	16 (57)	7 (44)
Rehabilitation physician, *n (%)*	9 (32)	3 (19)
Physician intellectual disability medicine, *n (%)*	0	0
Homecare, *n (%)*	0	0
Paramedics, *n (%)*	0	0

* This column shows the number of notes in which the pACP characteristic from the left column is found

** This column shows the number of patients in which the pACP characteristic from the left column is found at least once

Only 22% of the notes (*n* = 28 notes) containing pACP elements were shared with other HCPs through medical letters, and elements were often described in less detail in letters than in the corresponding notes. At the level of the patients, pACP elements were shared with other HCPs in the form of a medical letter for 75% of the patients (*n* = 12 patients). The letters sent within the organization were addressed exclusively to medical specialists (*n* = 12 notes, 43%), while inter-organizational letters were primarily sent to general practitioners (*n* = 25 notes, 89%), pediatricians (*n* = 16 notes, 57%), and rehabilitation physicians (*n* = 9 notes, 32%). The letters were never directed to allied healthcare or home care providers. No pACP elements were found in the incoming correspondence.

### Timelines

**Fig. 2:**
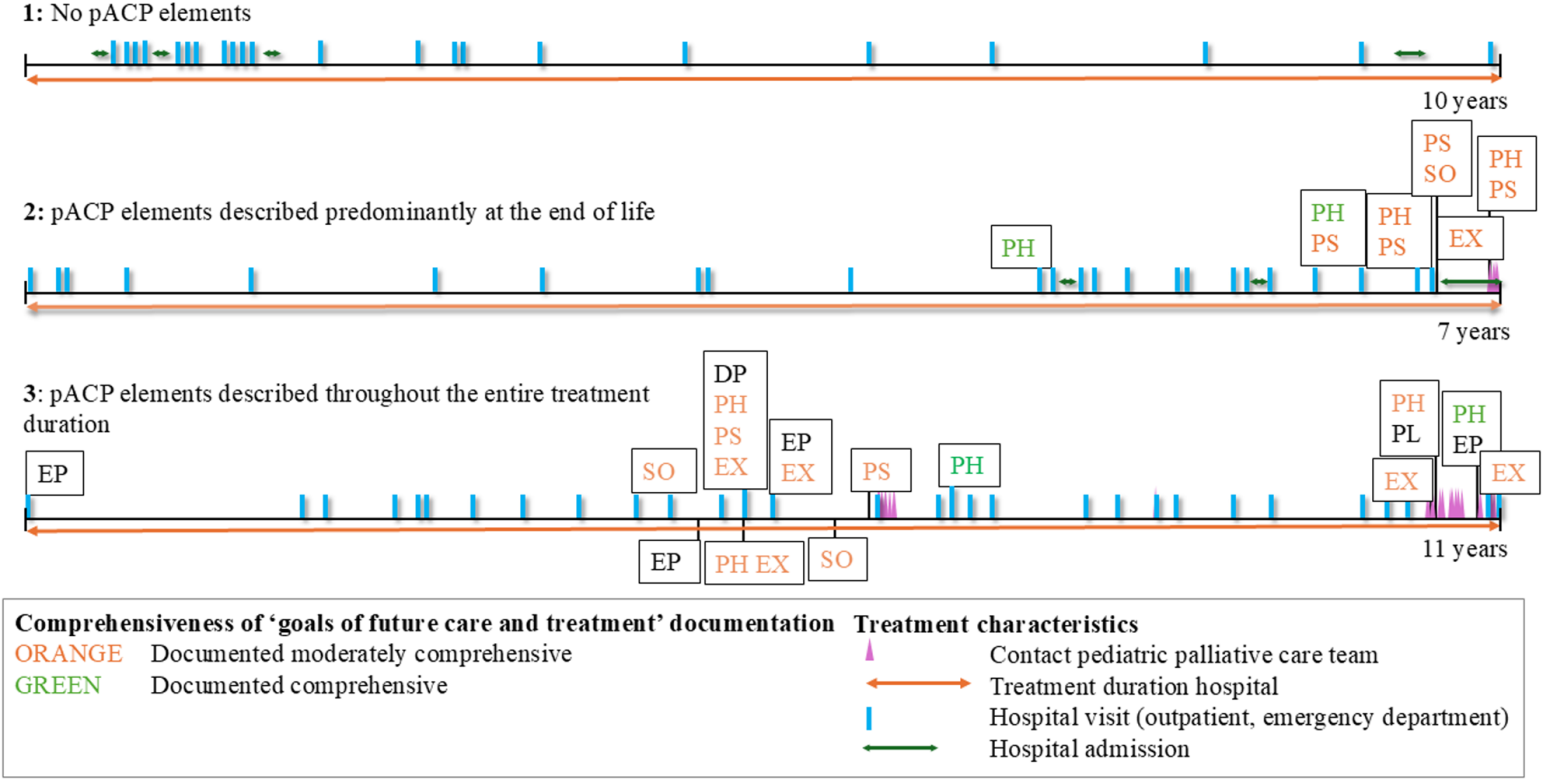
Illustrative timelines of distinct pACP trajectory patterns from first hospital contact to death DP = diagnostic and prognostic information; PH/PS/SO/EX = goals of future care and treatment (physical, psychological, social, existential); EP = emergency planning; PLE = preferred location of end-of-life care; PLD = preferred place of death

Illustrative timelines for each patient group are presented in Fig. 2.

### Qualitative component: structured interviews

#### Demographic characteristics

Seven primary physicians with different subspecialities (pediatric neurologist, pediatric intensivist, pediatric nephrologist, pediatrician congenital and hereditary disorders, general pediatrician, pediatrician metabolic disorders) were interviewed (*N* = 10) (Fig. 1). Their experience with care for children with LLCs varied from < 5 years (*n* = 2) to 20–30 years (*n* = 2). One primary physician was trained in pACP. When physicians were the primary physician for more than one of the included patients, they were interviewed about the different patients sequentially.

#### Content of conversations and documentation

In response to the individual pACP timelines, four out of seven (57%) primary physicians reported having discussed pACP elements more frequently than documented. For the domain-specific pACP timelines, six (86%) primary physicians reported discussing the physical domain more frequently, all (100%) reported discussing the psychological domain more frequently, and four (57%) reported discussing the social and existential domain more frequently than documented. An explanation for this discrepancy according to physicians was that they had a broader perspective on the definition of pACP than the researchers, who adhered strictly to the definition of pACP in the guidelines [10, 13]. For instance, while the researchers considered the future perspective as essential, the physicians also considered discussions about patients current situation to be part of ACP.

*“I think we*,* we’re getting into a culture where you increasingly call more advance care planning*,* right? And that you could put in the electronic health record*,* so to speak: Advance care planning. But advance care planning is discovering each other’s wishes and insights. And using that to follow a path to the future*,* after you’ve discussed multiple paths. And I think sometimes we discuss within physical domain or physical dimension very much the facts and then I wonder if it’s then seen by you as such. But that I might see it that way.” - pediatric intensivist*,* interview 6*.

An additional reason for the discrepancy mentioned by the physicians is that not all discussions involving pACP elements were documented, due to various factors. A first factor was responsibility. Primary physicians felt that it was their responsibility to ensure that the same information was not repeatedly documented in the electronic health records and that they should only document the physical domain as a physician. They believed documenting pACP elements was the responsibility of other HCPs.

*“But I can well imagine that occasionally there are just things in here where I thought ‘O*,* someone else will write that down.’ For example*,* here the neurologist wrote down another bunch. And then I think*,* there’s so much already written down*,* I’m not going to write it down again.” – pediatric nephrologist*,* interview 5*.

*“So I also think what one discusses*,* what the other discusses. Because we don’t all write down on repeat.” – pediatrician metabolic disorders*,* interview 3*.

Secondly, primary physicians highlighted the importance of determining what information is relevant for documentation. Certain discussions might not be considered relevant for other HCPs might be seen as too ‘soft’ for the electronic health record, especially discussions in the psychological or existential domain. One physician noted that the function of documentation was to account for the medical choices made.

*“I think the electronic health record is to at least write down core information so that*,* if someone else has to take care of the patient*,* that they can take over primary medical care*,* in my case. Electronic health records are also a kind of accountability*,* aren’t they? Diagnosis you often write down at length*,* therapy choices you write down at length. These kinds of softer things*,* which are probably much more important to people*,* I don’t make extensive records*,* so then you don’t come across them anyway.” – pediatric neurologist*,* interview 7*.

Finally, the complexity of documenting pACP elements was mentioned, underscoring the personal nature of the content and the challenges associated with preserving nuance into the documentation.

*“Well no*,* too much documentation is going to*,* I think*,* dumb down the content a bit too” – pediatric intensivist*,* interview 6*.

#### Sharing of pACP elements

Six (86%) primary physicians mentioned they had shared the pACP elements both intra- and inter-organizationally. Intra-organizationally, the most prevalent method for sharing was through verbal communication (80%), with the recipients primarily consisting of other physicians (80%), nurses (40%) and pediatric palliative care team members (40%). To a lesser extent, communication occurred with psychologists (20%), child life specialists (20%) and physiotherapists (20%).

Inter-organizationally, the most prevalent method for sharing was by telephone (80%) or medical letter (50%), with the recipients primarily consisting of the general practitioners (70%). However, home care providers (20%) and regional pediatricians (20%) were also mentioned as recipients.

The primary physicians stated that they did not consistently document the sharing of pACP elements with other HCPs, either due to time constraints or because they considered documentation necessary only when new agreements were made. Information regarding pACP communicated inter-organizationally was less detailed than information communicated intra-organizationally. Underlying factors according to physicians included concerns about the confidentiality and privacy in external communication, the relevance and importance of domains in external communication, and the methods of communication used.

*“You share depending on the situation with the person for who it is relevant.” - pediatric neurologist*,* interview 4*.

*“And that you know that parents are reading along*,* so your word choice is a bit different.” - Pediatrician*,* interview 1*.

#### Physician reported recommendations for practice

Physicians provided several recommendations during the interviews. They emphasized the need for more guidance on documenting pACP elements and proposed implementing a standardized electronic health record format, that could be based on the four domains of palliative care. They believed that this would facilitate both documentation and retrieval of information within the electronic health record.

*“Well*,* what I get out of this myself when I hear it like this*,* is that I like the idea of breaking it down into domains like this (physical*,* psychological*,* social*,* existential)” – general pediatrician*,* interview 1*.

*“Something with smart phrases*,* if you really want to find things back” – pediatrician metabolic disorders*,* interview 3*.

## Discussion

This study provides an overview of the extent to which documentation and sharing of pACP elements with other HCPs is implemented in current practice for children with LLCs. This retrospective medical chart review showed that approximately half of the children with LLCs had documented pACP elements in their electronic health records, and that these pACP elements were infrequently shared with other HCPs. In structured interviews, primary physicians of the included patients reported that they had discussed and shared pACP elements more often than was documented. The findings provide important insights for improving pACP documentation and sharing. These insights can be divided into four key points: (1) Steps in pACP documentation: identification, initiation, discussion and sharing; (2) Early initiation of pACP; (3) Documentation of all pACP domains and elements; (4) Consistent intra- and interprofessional sharing.

### Steps in pACP documentation: identification, initiation, discussion and sharing

Established guidelines [12–14] recommend *initiating* and *discussing* pACP with all patients with an LLC and/or their families and *documenting* and *sharing* pACP elements across the care network. These patients must first be *identified* as eligible for pACP. In our study, pACP elements were present in only half of the reviewed electronic health records. For the other patients it is unclear whether they were not identified as eligible for pACP, whether pACP was not initiated, whether there were other reasons for not discussing pACP elements, such as rejection by parents, or whether pACP was discussed but not documented.

The electronic health records lacked documentation of the various pACP steps (identification, initiation, discussion and sharing [10, 24]), thus providing little insight in the pACP process.

In the interviews, primary physicians mentioned several reasons for under-documentation of pACP including: wanting to avoid repetition, unclear responsibility for documentation, lack of perceived relevance to other HCPs and complexity of documentation. Several other factors could have played a role. First, for patients without documented pACP elements their primary physician was less frequently employed at the study hospital, and they often received less high-level care at the study hospital, demonstrated by the infrequent hospital visits in the final year of life, limited involvement of the pediatric palliative care team, and long gaps between last contact and death. As a consequence, there may have been less opportunities to initiate or discuss pACP. pACP elements may have been initiated or discussed in other settings and not shared with the study hospital, as no pACP elements were identified in the incoming correspondence. This might be the result of the pACP process in which it was decided to discontinue the treatment relationship with the study hospital due to treatment limitations. At the same time, pACP might be not addressed at all, for example due to unclear responsibility within the care network regarding initiating or discussing pACP elements. Second, the cause of death of patient without pACP elements was often undocumented or not in line with the underlying LLC. This might provide HCPs with little opportunity to anticipate the impending death and they might not feel the urge to initiate or discuss pACP elements, which is a well-known barrier to initiating pACP [9, 25–27].

It remains unclear whether any of the four pACP steps (identification, initiation, discussion and sharing) took place but were not documented, or whether they were not undertaken at all. In either case, if patient’s or family’s values, goals and preferences are not discussed, recorded or shared, care may not align with them. Even if pACP took place elsewhere, clinicians at the study hospital would lack the information needed to act accordingly. To close this gap, parents often share pACP themselves, although it is acknowledged that this is burdensome for them [13]. Thus, the documentation and sharing of each pACP step, when undertaken, helps involved HCPs align their care to the pACP process or steps.

### Early initiation of pACP

This study suggests that the timing of initiating pACP often does not align with the (inter)national guidelines and supporting literature, suggesting that the optimal timing for initiating pACP is during a stable phase of disease, shortly after diagnosis [11, 13, 27]. In this study, initiation of pACP elements occurred approximately one to five years after diagnosis, predominantly during a phase of deterioration. In the majority of patients, ‘provision of information on diagnosis and prognosis’ was the first pACP element documented. It is plausible that the discussion of diagnosis or prognosis prompted the consideration of other pACP elements at that time or at a later stage [28]. However, discussions regarding diagnosis and prognosis may have occurred more frequently than our results suggest as we only included them when accompanied by other pACP elements. Future studies could adopt a longitudinal approach to explore in more detail which pACP elements are discussed over time with pediatric patients with LLCs, and what factors triggers the initiation and repetition of these discussions.

The individual patient timelines visualized the different pACP trajectories over time, revealing a subgroup of patients for whom pACP was initiated only at end of life. It is interesting that this group of patients was older at the time of death and the pediatric palliative care team became involved at a later stage then the subgroup of patients with documented pACP elements throughout the entire treatment duration. This finding may be explained by the relatively recent implementation of pediatric palliative care teams and pACP in the Netherlands. The late initiation of pACP in the course of disease can result in care that is incongruent with the values, goals and preferences of the patient and their family for an extended period [26]. Therefore, it is important to direct attention towards the further implementation of pACP as standard care early in the course of disease.

### Documentation of all pACP domains and elements

There is potential for improving the comprehensiveness of pACP documentation, with sufficient attention to all relevant elements and domains. Documented pACP elements in this study predominantly included ‘goals of future care and treatment’, while those pertaining to ‘preferred location for end-of-life care’ or ‘preferred location for death’ were least frequently documented. A possible explanation is that the former encompasses a more extensive range of subjects while the latter two elements are more specifically defined. In addition, earlier studies found that the future death of the child is a sensitive topic to discuss for both parents and HCPs, yet it is a concept that parents often already have in mind [29, 30]. Moreover, prior research indicates that when such topics are discussed at an early stage, the child is more likely to die in the preferred location [31], while also enabling parents to experience a greater sense of control by preparing for the future [5]. At the same time, it has been observed that parents often prefer to leave all options open rather than making a definitive decision, for example about location of death [32]. It is possible that this topic was discussed, but no concrete conclusions were reached, which may explain why it was not documented. This aligns with literature suggesting that healthcare providers focus on documenting specific agreements from pACP discussions, while parents prioritize recording the context and process [6, 33]. Another reason for the limited documentation of these end-of-life topics may be the difficulties primary physicians reported in documenting sensitive topics within electronic health records due to their complexity. Simultaneously, discussing these topics with parents in advance can be helpful, as it prepares them to make informed decisions later, if and when the need arises [5]. Education and guidance can support HCPs in early discussion and documentation of ‘preferred location for end-of-life care’ and ‘preferred location for death’. In the Netherlands, the IMPACT toolkit [34, 35] was developed to give guidance and training to a range of HCPs in pACP. It is expected that this will contribute to the improved discussion of these topics in the future.

Within the pACP process, the goals of future care and treatment are explored across the physical, psychological, social and existential domains. This study found the physical domain to be the most frequently documented, which is in line with literature suggesting that HCPs and parents prioritize medical issues [2, 7]. However, different pACP tools such as the IMPACT toolkit [34, 35] help HCPs giving attention to all the four domains by exploring the values of the patient and/or their family as a starting point of the pACP process. The extent to which these pACP tools were used within the reviewed patients in the current study is not known. Interviewed primary physicians reported focusing on the physical domain when documenting pACP in the electronic health record, assuming that documentation of other domains was the responsibility of other HCPs. As a result, what is documented about pACP does not accurately reflect the content of the discussions that took place, and the other domains might have been discussed more often than found in the medical record. Since the Dutch guideline does not provide clear direction on the content of documentation, it would be beneficial to expand the advice. Recommendations should be based on the preferences of parents and HCPs, providing clearer guidance on what should be documented as a minimum, as also suggested by the interviewed primary physicians. This will be taken into account during the further development of the IMPACT toolkit.

### Consistent intra- and interprofessional sharing

Finally, our results on the inter- and intra-organizational sharing of pACP elements shows room for improvement. The majority of the notes containing pACP elements were not shared with other HCPs through medical letters. It is important to notice that documentation in the electronic health record is a form of intra-organizational sharing in a passive way, as the recipient must actively search for the information recorded, unlike more direct forms of communication, such as phone calls or in-person discussions. Primary physicians in the interviews asserted that pACP elements are more frequently shared than could be ascertained through the electronic health records, predominantly through verbal communication. Children with LLCs often have complex care networks, including involvement of many HCPs working at different organizations without interprofessional relations and HCPs that incidentally replace the predominantly involved HCPs, for example during night shifts [36, 37]. In consideration of these complex networks, it is reasonable to hypothesize that if the pACP elements are only communicated verbally, a significant proportion of the network remains unaware of them. The documentation of these elements could form as basis for sharing these with all the involved HCPs. Given that physicians primarily share pACP elements verbally, developing a standardized interprofessional communication strategy could enhance information sharing among HCPs, within standardized multidisciplinary team meetings as one possible approach. This would ensure that both directly involved physicians, and other key HCPs are adequately informed. Additionally, as stated before, clearer guidance and structured frameworks could provide direction for HCPs in documenting pACP.

Another interesting finding is that the recipients of the medical letters were predominantly general practitioners, while it is known that the general practitioner does not always play an active role in the care network of children with LLCs [38–40]. This presents an opportunity to involve general practitioners in the pACP process at an earlier stage, as they could identify palliative patients or families caring for children with LLCs. Empowering them to take a proactive role in pediatric palliative care could enhance family support and facilitate access to valuable resources [40]. Furthermore, enhancements should be made by identifying other key HCPs within the complex care networks and ensuring that information is consistently sent to them. It is important to take into account the regulations surrounding data sharing, physician-patient confidentiality and privacy, which may impose constraints on the extent to which information can be disseminated to all HCPs.

Notably, the pediatric palliative care team was the documented author in only 9% of all pACP notes, despite often having a clear overview of involved healthcare providers [41]. This is an interesting finding, as pediatric palliative care team members [41, 42] are well positioned to assume greater responsibility in discussing, documenting, and sharing ACP elements, which could improve coordination in these complex care networks.

### Strengths and limitations

The major strength of this study is that an explanatory sequential design was adopted, which offered the advantage of providing a multi-source perspective on the data. When the medical chart review had been conducted in isolation, results would have been subject to bias, as the documentation of pACP elements in the records likely would have underestimated the extent to which these elements were actually discussed. This bias would most likely have arisen from the fact that HCPs do not always document all pACP discussions, as found in the interviews. Conversely, relying solely on the primary physician self-reported information risked recall bias, compounded by the known tendency of HCPs to overestimate positive aspects of their own practice [43]. By combining the qualitative and the quantitative results, we aimed to achieve a more comprehensive and realistic understanding of pACP documentation and sharing in current practice, while also identifying current main barriers and facilitators involved.

The study also had some limitations. Firstly, it is a single-center study with a small sample of deceased patients, which restricts the generalizability of its findings to other hospitals. Secondly, a limitation of this study is that solely primary physicians of patients with documented pACP elements in their electronic health records were selected for interviews. The results demonstrated that physicians recalled events differently than to what is reflected in the medical chart review. Therefore, it might have been valuable to explore what the primary physicians and other involved HCPs of the patients without documented pACP elements would report about the medical chart review findings. In line with this, it would also have been valuable to ask the parents of patients with and without documented pACP elements about their perspectives on the course of care. However, due to time constraints, the scope of this study, and concerns about burdening these parents, we were unable to include these additional participants.

### Further research

Further research should further explore the barriers and facilitators regarding the documentation of pACP discussions and sharing these pACP elements in order to gain a more comprehensive understanding of determinants influencing the implementation of pACP. This could be achieved by exploring the perspectives of parents, children with LLCs and a broader range of HCPs on documentation and sharing of pACP elements and wishes for implementation in clinical practice. Another form of contributory research would be a comprehensive review of the existing literature on documentation and sharing of the ACP process. These studies should be undertaken prior to designing a more extensive guideline on the documentation and sharing of pACP. The findings from these studies will also be integrated into the IMPACT toolkit, which will provide healthcare professionals with training on best practices for pACP documentation and sharing.

## Conclusion

This mixed-method study evaluated for the first time the documentation and inter-professional sharing of pACP in the care for children with life-limiting conditions in the Netherlands. A medical chart review showed that pACP outcomes were documented for approximately half of the deceased children, with variation in the type of pACP elements documented, and limited documentation of pACP outcomes being shared with other healthcare professionals. Interviews with primary physicians highlighted a discrepancy between actual care and documentation, as they reported higher frequency of pACP discussions and sharing than was documented. These results indicate a need for improved pACP implementation, with a focus on timely initiation of pACP, comprehensive documentation of all pACP steps (identification, initiation, discussion and sharing), elements and domains, and adequate sharing across the care network. A prospective study directly comparing pACP discussions with the documentation would help to verify the gap between care and documentation observed in this study. Additionally, further research is needed to identify barriers and facilitators to documentation and sharing, and explore the preferences of children, families, and HCPs regarding pACP.

## Supplementary Information

Supplementary Material 1.

Supplementary Material 2.

Supplementary Material 3.

Supplementary Material 4.

## Data Availability

The data of this study are kept by ST in the Radboud university medical center, Nijmegen, The Netherlands, and are available upon reasonable request.

## Abbreviations

pACP: pediatric advance care planning
LLC: life limiting condition
HCP: health care professional
