# Supplementary material for: Pediatric advance care planning: a mixed-methods evaluation of documentation and sharing in current practice
Source: BMC Palliat Care. 2026 Jan 29;25:51. doi: 10.1186/s12904-026-01992-7 (PMC12924617; doi:10.1186/s12904-026-01992-7)
Supplement: Supplementary file 1 — Supplementary Material 1. [file 12904_2026_1992_MOESM1_ESM.docx]

**Supplementary material 1. Data extraction protocol**

1. Patient characteristics
   1. Age at diagnosis
   2. Age at death
   3. Biological sex
2. Disease and provided care characteristics
   1. Main diagnosis
   2. Cause of death
   3. Location of death
   4. Hospital visits
   5. Primary physician
   6. Expertise of primary physician
   7. Work experience primary physician
   8. pACP training primary physician
   9. Care network
   10. Involvement pediatric palliative care team
   11. Repeating data – notes pediatric palliative care team
       1. Date
       2. Type of contact
3. pACP elements
   1. Repeating data – notes with elements of pACP
      1. Date
      2. Persons present
      3. Leader of conversation
      4. Stage of disease
      5. Location of conversation
      6. Elements of pACP
      7. Dimensions of pACP
         - Comprehensiveness
      8. Medical letter
         - Comprehensiveness
         - Recipients
      9. Individual care plan
