# Supplementary material for: Pediatric advance care planning: a mixed-methods evaluation of documentation and sharing in current practice
Source: BMC Palliat Care. 2026 Jan 29;25:51. doi: 10.1186/s12904-026-01992-7 (PMC12924617; doi:10.1186/s12904-026-01992-7)
Supplement: Supplementary file 2 — Supplementary Material 2. [file 12904_2026_1992_MOESM2_ESM.docx]

**COREQ (Consolidated criteria for Reporting Qualitative research) Checklist**

A checklist of items that should be included in reports of qualitative research. You must report the page number in your manuscript where you consider each of the items listed in this checklist. If you have not included this information, either revise your manuscript accordingly before submitting or note N/A.

| **Topic** | **Item No.** | **Guide Questions / Description** | **Reported on Page No.** |
| --- | --- | --- | --- |
| **Domain 1: Research team and reflexivity** | | | |
| *Personal characteristics* | | | |
| Interviewer/facilitator | 1 | Which author/s conducted the interview or focusgroup? | EV - *page 11* |
| Credentials | 2 | What where the researcher’s credentials? E.g. PhD, MD | EV Bachelor’s degree with supervision of ST, MD and PhD candidate - *page 11* |
| Occupation | 3 | What was their occupation at the time of the study? | EV (Medical Student); ST (Physician and PhD candidate) – page 11 |
| Gender | 4 | Was the researcher male or female? | Female |
| Experience and training | 5 | What experience or training did the researcher have? | EV and ST got training in qualitative research with supervision of ED, postdoctoral researcher with more experience in qualitative research – *page 11* |
| *Relationship with participants* | | | |
| Relationship established | 6 | Was a relationship established prior to study commencement? | No participants were known to EV. – *page 11* |
| Participant knowledge of the interviewer | 7 | What did the participants know about the researcher? e.g. personal goals, reasons for doing the research | EV is a medical student. |
| Interviewer characteristics | 8 | What characteristics were reported about the interviewer/facilitator? e.g. Bias, assumptions, reasons and interests in the research topic | EV is a medical student. – *page 11* |
| **Domain 2: Study design** | | | |
| *Theoretical framework* | | | |
| Methodological orientation | 9 | What methodological orientation was stated to underpin the study? e.g. grounded theory, discourse analysis, ethnography, phenomenology, content analysis | Inductive approach – *page 11* |
| *Participant selection* | | | |
| Sampling | 10 | How were participants selected? e.g. purposive, convenience, consecutive, snowball | Purposive – *page 10* |
| Method of approach | 11 | How were participants approached? e.g. face-to-face, telephone, mail, email | E-mail – *page 10* |
| Sample size | 12 | How many participants were in the study? | Seven – *page 12* |
| Non-participation | 13 | How many people refused to participate or dropped out? Reasons? | Six, due to no employment at the study hospital (n=2) or lack of time to schedule the interview (n=4) – *page 13* |
| *Setting* | | | |
| Setting of data collection | 14 | Where was the data collected? e.g. home, clinic, workplace | In-hospital – *page 11* |
| Presence of non-participants | 15 | Was anyone else present besides the participants and researchers? | No interpreter required |
| Description of sample | 16 | What are the important characteristics of the sample? e.g. demographic data, date | Being a primary physician – *page 10* |
| *Data collection* | | | |
| Interviewguide | 17 | Were questions, prompts, guides provided by the authors? Was it pilot tested? | Attached (Additional file 3) |
| Repeat interviews | 18 | Were repeat interviews carried out? If yes, how many? | No |
| Audio/visual recording | 19 | Did the research use audio or visual recording to collect the data? | Audio recording using Microsoft Teams – *page 11* |
| Field notes | 20 | Were field notes made during and/or after the interview or focus group? | No |
| Duration | 21 | What was the duration of the interviews or focus group? | 30-60 minutes *– page 11* |
| Data saturation | 22 | Was data saturation discussed? | No |
| Transcripts returned | 23 | Were transcripts returned to participants for comment and/or correction? | No |
| **Domain 3: Analysis and findings** | | | |
| *Data analysis* | | | |
| Number of data coders | 24 | How many data coders coded the data? | Two, EV and ST *– page 11* |
| Description of coding tree | 25 | Did authors provide a description of the coding tree? | Yes – *Page 11 and Additional file 4* |
| Derivation of themes | 26 | Were themes identified in advance or derived from the data? | Derived from the data – *page 11* |
| Software | 27 | What software, if applicable, was used to manage the data? | Microsoft Word and Microsoft Excel |
| Participant checking | 28 | Did participants provide feedback on the findings? | No |
| *Reporting* | | | |
| Quotations presented | 29 | Were participant quotations presented to illustrate the themes/findings? Was each quotation identified? e.g. participant number | Yes, see Additional file 4 and *page 19 - 22* |
| Data and findings consistent | 30 | Was there consistency between the data presented and the findings? | Quotations provided to illustrate each thema - *page 19-22* |
| Clarity of major themes | 31 | Were major themes clearly presented in the findings? | Yes – p*age 19-22* |
| Clarity of minor themes | 32 | Is there a description of diverse cases or discussion of minor themes? | Yes – p*age 19-22* |

Developed from: Tong A, Sainsbury P, Craig J. Consolidated criteria for reporting qualitative research (COREQ): a 32-item checklist for interviews and focus groups. International Journal for Quality in Health Care. 2007. Volume 19, Number 6: pp. 349 – 357

**Once you have completed this checklist, please save a copy and upload it as part of your submission. DO NOT include this checklist as part of the main manuscript document. It must be uploaded as a separate file.**
