# Supplementary material for: Pediatric advance care planning: a mixed-methods evaluation of documentation and sharing in current practice
Source: BMC Palliat Care. 2026 Jan 29;25:51. doi: 10.1186/s12904-026-01992-7 (PMC12924617; doi:10.1186/s12904-026-01992-7)
Supplement: Supplementary file 3 — Supplementary Material 3. [file 12904_2026_1992_MOESM3_ESM.docx]

**Supplementary material 3. Structured interviewguide**

1. Introduction

*Show individual patient pACP trajectory in timeline*

1. Key elements of advance care planning
   1. Goals and preferences for future care and treatment
   2. Acute medical scenarios in the future
   3. Preferences for location for end-of-life care
   4. Preferences for location for dying
2. Moments of advance care planning
3. Content of advance care planning conversations
   1. Medical dimension
   2. Psychological dimension
   3. Social dimension
   4. Existential dimension
4. Documentation of advance care planning
5. Complete trajectory of advance care planning
   1. Other healthcare professionals
   2. Healthcare professional discussing advance care planning
   3. Way of receiving information
   4. Process information
6. Sharing of outcomes
   1. Healthcare professionals (intramural versus extramural)
   2. Methods of sharing
   3. Dimensions shared
   4. Differences
