# Supplementary material for: Pediatric advance care planning: a mixed-methods evaluation of documentation and sharing in current practice
Source: BMC Palliat Care. 2026 Jan 29;25:51. doi: 10.1186/s12904-026-01992-7 (PMC12924617; doi:10.1186/s12904-026-01992-7)
Supplement: Supplementary file 4 — Supplementary Material 4. [file 12904_2026_1992_MOESM4_ESM.docx]

| **Documentation of pACP elements** | | | |
| --- | --- | --- | --- |
| **Theme** | **Subtheme** | **Code** | **Example Quote** |
| Discrepancy in definition ACP physician versus researcher | Difference in definition ACP physician versus researcher | Difference in definition ACP physician versus researcher | *I think we, we're getting into a culture where you increasingly call it advance care planning, right? And that you could put in the patient file, so to speak: Advance care planning is discovering each other's wishes and insights. And using that to chart a path to the future, after you've discussed multiple paths. And I think sometimes we discuss within medical domain or medical dimension very much it's about facts and then I wonder if it's then seen by you as such. But that I might see it that way then.* |
|  |  | Healthcare provider believes that ACP covers more than what is actually stated in the record. | *We have discussed nutrition on numerous occasions. I am unaware of your search criteria, but if you search for nutrition or tube feeding, I believe you will find a significant amount of information. I have never included the term advance care planning, so that could be the reason.* |
| Physician did not document everything | Responsibility | Documentation of ACP was done by other HCP | *But I can well imagine that occasionally there are just things through here that I thought ‘Oh, someone else will write that down.’ For example, here the neurologist wrote down another bunch. And then I think, there's so much already written down, I'm not going to write it down again.* |
|  |  |  |  |
|  |  | Notes of an physicians should (only) contain medical information |  |
|  |  | To prevent repetition of already known information in the EHR |  |
|  |  |  | *Yes, I think so, broadly speaking, although with other patients, of course, that care is shared within a team, with everyone taking turns to make notes. And also taking turns to play a leading role in that. Depending on which topic needs more attention at that moment, or is more medical in nature, or more supportive in other ways, such as logistics.* |
|  |  | Division of tasks within the healthcare team |  |
|  |  |  |  |
|  |  | Asking after this dimension is standardized procedure/work |  |
|  | Relevancy | Physician thought it not relevant for other HCP | *I think that when I have an outpatient clinic visit, certainly with this family, it would come up, but not to the extent that I would write it down. You do ask how the other children are doing, but I don't think it's necessary to write that down.* |
|  |  | Since this subject is something 'soft' |  |
|  |  | The intention of the conversation was solely for the physician and not other HCP |  |
|  |  | The information was obtained by intuition of the physician | *I think the electronic health record is to at least write down core information so that, if someone else has to take care of the patient, that they can take over primary medical care, in my case. Medical records are also a kind of accountability, aren't they? Diagnosis you often write down at length, therapy choices you write down at length. These kinds of softer things, which are probably much more important to people, I don't make extensive records, so then you don't come across them anyway.* |
|  |  | Since this subject is not of use in the accountability of medical decisions |  |
|  |  | If a physicians knows the family well, and vice versa, then the physicians feels less tendency to account for some decisions |  |
|  | Complexity | Documenting this will simplify the content of the dimension | *Well no too much documentation is going to, I think, dumb down the content a bit too* |
|  |  | Situation is too complex to write it down |  |
|  |  | Personal information is not documented |  |
| **Sharing of pACP elements** | | | |
| **Theme** | **Subtheme** | **Code** | **Example Quote** |
| Barriers in sharing of pACP elements | Time constraints | Sharing information external requires more effort | *Since extramural you have to do your best to hand over knowledge.* |
|  | Only new agreements have to be shared | Main focus on sharing agreements | *I can imagine that you do not write down everything as comprehesive as you discussed it, but you do (write down) the final agreements.* |
| Differences in intra- and inter-organizational communication | Confidentiality and privacy concerns | Personal information of the parents or patient should not always be shared with everyone | *No difference if I hand over the care, but it differs if it is just for information, than I find some things personal. And then I think that not necessarily concerns all the clinics… And if a letter is sense to everybody, than I try to respect the privacy of the parents, by considering what I write in it* |
|  |  | The way of communication (oral vs written) has impact on the content and the comprehensiveness |  |
|  |  | Physician is more critical in what is shared with external HCP, since nuance to personal information can be lost | *And that you know that parents are reading along, so your word choice is a bit different.* |
|  |  | Parents reading along affects the content |  |
|  | Order in importance in dimensions in communication | Every dimension is shared extramural, but the medical dimensions is the most important | *At first instance the medical, but the psychological and the spiritual and social things depend on this.* |
|  |  | The spiritual dimension is subordinate in sharing both intra- and extramural |  |
|  | The relevance of the information for external HCP | Relevance of the information according to the physician | *The threshold is then, it should really be relevant, since extramural you have to do your best to hand over knowledge. I think it is more important that they keep him alive and cure him, than that they know that his mom is a (job mother).* |
|  |  | In complex and academic cased the GP is less involved |  |
